# How do medical doctors use a web-based oncology protocol system? A comparison of Australian doctors at different levels of medical training using logfile analysis and an online survey

**DOI:** 10.1186/1472-6947-13-82

**Published:** 2013-08-04

**Authors:** Julia M Langton, Bianca Blanch, Nicole Pesa, Jae Min Park, Sallie-Anne Pearson

**Affiliations:** 1 Faculty of Pharmacy, The University of Sydney, Sydney, NSW, Australia; 2 Faculty of Medicine, The University of New South Wales, Sydney, NSW, Australia

**Keywords:** Clinical decision support systems, Evidence-based practice, Medical education, Cancer chemotherapy protocols, Health personnel, ‘Medical staff, Hospital’

## Abstract

**Background:**

Electronic decision support is commonplace in medical practice. However, its adoption at the point-of-care is dependent on a range of organisational, patient and clinician-related factors. In particular, level of clinical experience is an important driver of electronic decision support uptake. Our objective was to examine the way in which Australian doctors at different stages of medical training use a web-based oncology system (http://www.eviq.org.au).

**Methods:**

We used logfiles to examine the characteristics of eviQ registrants (2009–2012) and patterns of eviQ use in 2012, according to level of medical training. We also used a web-based survey to evaluate the way doctors at different levels of medical training use the online system and to elicit perceptions of the system’s utility in oncology care.

**Results:**

Our study cohort comprised 2,549 eviQ registrants who were hospital-based medical doctors across all levels of training. 65% of the cohort used eviQ in 2012, with 25% of interns/residents, 61% of advanced oncology trainees and 47% of speciality-qualified oncologists accessing eviQ in the last 3 months of 2012. The cohort accounted for 445,492 webhits in 2012. On average, advanced trainees used eviQ up to five-times more than other doctors (42.6 webhits/month compared to 22.8 for specialty-qualified doctors and 7.4 webhits/month for interns/residents). Of the 52 survey respondents, 89% accessed eviQ’s chemotherapy protocols on a daily or weekly basis in the month prior to the survey. 79% of respondents used eviQ at least weekly to initiate therapy and to support monitoring (29%), altering (35%) or ceasing therapy (19%). Consistent with the logfile analysis, advanced oncology trainees report more frequent eviQ use than doctors at other stages of medical training.

**Conclusions:**

The majority of the Australian oncology workforce are registered on eviQ. The frequency of use directly mirrors the clinical role of doctors and attitudes about the utility of eviQ in decision-making. Evaluations of this kind generate important data for system developers and medical educators to drive improvements in electronic decision support to better meet the needs of clinicians. This end-user focus will optimise the uptake of systems which will translate into improvements in processes of care and patient outcomes.

## Background

Evidence-based practice is the cornerstone of modern medicine. Rapid advances in medicine and information technology have provided the necessary impetus for the development and deployment of electronic decision support systems [1-3]. These systems synthesise large bodies of evidence, a task beyond that of individual clinicians. Electronic decision support systems have grown in popularity, play an important role in providing up-to-date resources for point-of-care use, and have been demonstrated to improve processes of medical care and patient outcomes [4-7].

Despite the significant benefits of electronic decision support, its adoption has been highly variable. Well-documented barriers to access are organisational, provider and patient-related [6,8-10]. Importantly, there are concerns on the part of clinicians, particularly doctors, that reliance on such systems may lead to deskilling in decision-making. Moreover, many experienced clinicians report their practices align with best evidence and that electronic decision support threatens professional autonomy. Conversely, these systems are more likely to be used when clinicians perceive they enhance decision-making and improve practice. As the medical profession becomes more technologically savvy and the culture continues to embrace the necessity to have the most up-to-date evidence at clinicians’ finger tips, some of these traditional barriers may carry less importance. These changes are also likely to be more apparent in medical specialties where new technologies and treatments are changing rapidly and there is a high risk of adverse patient outcomes.

Oncology practice is particularly demanding due to the complex nature of care and the challenges of achieving the delicate balance between maximising treatment effects and minimising toxicity [11,12]. As such, medical oncology like many other medical specialties has taken advantage of web-based technology by developing online guideline and protocol systems to support clinicians in their day-to-day practice [13]. However, the rapid proliferation of these online systems globally has not been accompanied by comprehensive evaluation of their use and impact in cancer treatments settings.

We have developed a multi-faceted research program evaluating an Australian web-based oncology system, eviQ treatments online [13-18]. We have demonstrated high rates of eviQ adoption by all cancer care health professionals but the nature and extent of use is highly dependent on clinicians’ specific roles in cancer care [14]. Our interview-based study of oncology practitioners found that junior cancer clinicians accessed eviQ more frequently than their senior counterparts. This finding is driven mainly by levels of familiarity with treatment practices; many senior doctors felt their experience negated the necessity to refer to the protocol system while junior doctors relied heavily on the program to guide decision-making [15,16]. Importantly, junior doctors were more inclined to embrace information technology than their senior colleagues and felt that eviQ gave them a greater sense of autonomy in their day-to-day practice.

Our aim is to expand and strengthen our previous research by examining how medical doctors at different stages of training use eviQ. Specifically, we will examine:

1) Characteristics of medical doctors registered on eviQ;

2) Patterns and frequency of eviQ use (logfile analysis) according to level of medical training and years of oncology experience; and

3) Doctors’ perceptions about how eviQ is used in clinical practice (online survey) and how responses of clinicians in training compare with speciality-qualified doctors.

## General methods

### Study setting

In Australia, cancer services are funded primarily by Australia’s universal health care funding arrangements. Medical and radiation oncology therapies are mostly delivered in the ambulatory care (outpatient) setting at metropolitan hospitals (university-affiliated, tertiary referral centres covering geographic areas of around 75 square kilometres), regional centres (with catchments up to 1,200 square kilometres) and rural hospitals (with catchments up to 3,400 square kilometres). Australia has over 700 public hospitals with a total of approximately 55,000 beds [19].

### eviQ cancer treatments online

eviQ (http://www.eviQ.org.au) is a web-based oncology protocol system managed by the Cancer Institute New South Wales (NSW), a government funded agency established to improve cancer control. eviQ primarily targets health professionals involved in implementing cancer care by providing detailed and extensive instructions on how to deliver evidence-based treatments safely and appropriately. Treatment information encompasses adolescent and young adult care, cancer genetics, haematology, haemapoietic progenitor cell transplants, medical oncology, nursing, primary health, palliative care and radiation oncology. The site comprises over 1,300 protocols, developed by a consensus process involving specialist physicians, nurses, pharmacists and allied health practitioners from across Australia. Each protocol undergoes a comprehensive review every 1 to 2 years. While the primary eviQ target audience is health professionals, the site also publishes information tailored specifically to cancer patients and their carers.

Until October 2009 eviQ was known as the Cancer Institute Standard Cancer Treatments Program (CI-SCaT); the system underwent a major rebuild and rebranding to better meet the specific needs of health professionals and patients. The previous platform (CI-SCaT) was taken offline on March 31 2010 to allow sufficient opportunity for users to transition and register on the eviQ website.

### Medical training in Australia

In Australia, medical training generally occurs according to the following pathway; completion of a medical degree (4 to 6 years duration); at least 2 years of general medical training as an ‘intern/resident’; at least 2 years training as a ‘registrar’; and training for at least 3 years as an ‘advanced trainee’ (most of this time is focused on the doctors chosen medical speciality such as medical oncology, haematology). Doctors are specialty-qualified at the completion of their advanced training, at which time they become ‘fellows’ and ‘staff specialists’.

Doctors can be exposed to oncology practice from the time of internship when they may rotate through the specialty as part of their general medical training. Advanced trainees choosing an oncology specialty will practice almost exclusively in this field; will most often be the key interface between patients and the treating medical team; and will also supervise more junior medical staff. Staff-specialists are ultimately responsible for the medical team and treatment decisions.

### Ethical approval

This study was approved by the Population and Health Services Research Ethics Committee (HREC/10/CIPHS/70). We sought a waiver of consent to undertake the logfile analysis. We obtained informed consent from cancer clinicians for the completion of the online survey.

## Phase 1: eviQ logfile analysis

### Methods

#### Study design

We conducted a retrospective study of Australian medical doctors registered on eviQ between October 2009 (eviQ launch date) and December 2012. We examined registrant details and patterns of use from web-logfiles generated from the eviQ platform in 2012. Specifically, we compared eviQ use in doctors at various stages of medical training, from intern/resident through to the most senior doctors, staff specialists.

#### Data sources and analysis

The eviQ secretariat provided the research team with access to data from the eviQ platform. Demographic registrant and logfile data were obtained on-site at the Cancer Institute NSW in unit record format (stripped of personal identifiers such as usernames). The eviQ platform has the capacity to generate data on the characteristics of all registered users including registrant type (individual clinician or unit registration), role (e.g., medical, nursing, pharmacy), health sector (public, private, or both), main area of work (e.g., medical oncology, haematology), geographical location of practice, years of oncology experience, and source of referral to the eviQ website. This information is reported by users upon registration and website registrants are prompted to update this information on an annual basis.

The eviQ platform also generates data on logfiles that monitor webhits, defined as one click anywhere on the eviQ website. Logfiles can be stratified by any of the aforementioned variables (e.g., clinician type, years of oncology experience) and by the time at which the webhits occur (e.g., time of day, month, year). However, the current eviQ logfile reports are aggregated and do not have the capacity to examine clinician-level use. Further, our inability to examine variability by clinician limits any statistical analyses.

#### Study cohort (Figure 1)

**Figure 1:**
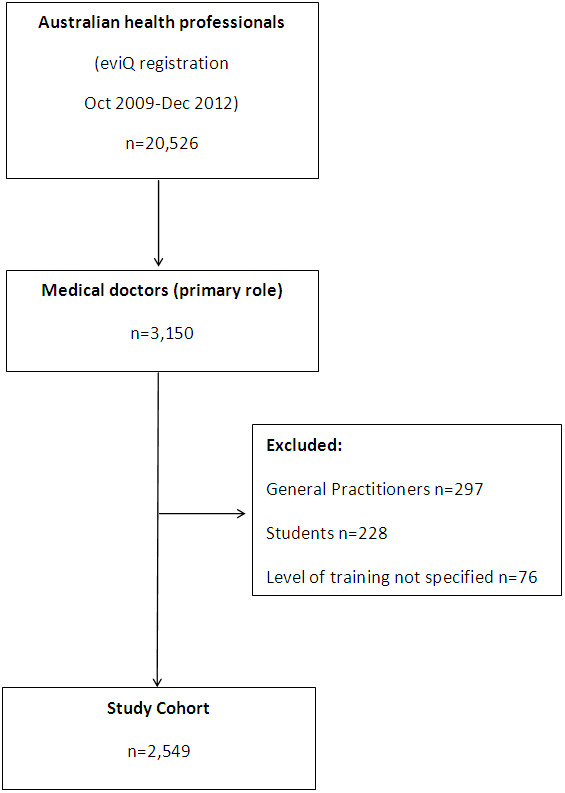
Flowchart of study cohort derivation.

Our cohort included Australian health professionals registered as individual clinicians and identifying themselves as medical doctors upon eviQ registration (Figure 1); approximately 8% of all registrations are medical units [14]. Medical unit registrations were excluded from our current analysis as they are likely to represent the web-activity of a group of health professionals with varying roles and years of clinical experience. We also excluded medical students, doctors whose level of training could not be determined, and doctors unlikely to be practicing in the hospital setting (those identifying as general practitioners or primary care physicians).

#### Data analysis and reporting

We used Microsoft Excel for logfile analyses and report on the following for our study cohort:

##### Registrant characteristics

We describe the registrant characteristics of our study cohort including level of training (interns/residents, registrars, advanced trainees, fellow or staff specialists), main area of work, year of registration, years of oncology experience, health sector, location (NSW or another Australian state), and eviQ referral source. We also report on recency of eviQ use in order to distinguish between frequent and infrequent eviQ users (login in the last 3 months, 6 months or 12 months of 2012).

We present this data for all medical doctors in our cohort and by level of training; interns/residents, registrars, advanced trainees, fellows, or staff specialists.

##### Patterns of eviQ use

We describe the patterns of eviQ use for our study cohort using logfiles generated in 2012. Importantly, we examined patterns of use from the launch of eviQ in October 2009 through to December 2012 and noted no trends in use over time with the exception of reductions in use over the Australian holiday periods (December, January). As such, the current manuscript focused solely on logfiles from the most recent year, 2012.

We report webhits by month (January-December 2012) using two metrics: raw webhits and rates (defined as webhits/100 registered medical doctors). The denominator for our rate analysis was the number of medical doctors who accessed the website at least once in 2012; approximately 35% of registrants did not use eviQ in 2012.

We stratified our analyses by the following:

•Level of training: interns/residents, registrars, advanced trainees, fellows, and staff specialists.

•Years of oncology experience: <2, 2–5, 5–10, and >10 years (categories pre-defined by eviQ website).

We also analysed raw webhits and rates of use by time of day and day of the week. Our previous logfile analyses of all Australian health professionals demonstrated that 87% of eviQ activity occurred during standard clinic hours (08:00–18:00, Monday through Friday) [14]. We examined whether similar patterns are observed in medical doctors overall and by level of training.

### Results

#### Registrant characteristics (Figure 1, Table 1)

At December 2012, there were 20,526 Australian health professionals registered on eviQ 3,150 (15.3%) of whom were medical doctors. After excluding general practitioners, students, and registrants not specifying their level of training, our cohort consisted of 2,549 medical doctors (Figure 1).

**Table 1:** Characteristics of study cohort

	**Total**		**Intern/Resident**		**Registrar**		**Advanced trainee**		**Fellow**		**Staff specialist**	
	**(N = 2549)**		**(n = 549)**		**(n = 669)**		**(n = 358)**		**(n = 135)**		**(n = 838)**	
**Main area of work**	**n**	**%**	**n**	**%**	**n**	**%**	**n**	**%**	**n**	**%**	**n**	**%**
Medical oncology	879	34.5	207	37.7	190	28.4	145	40.5	40	29.6	297	35.4
Haematology	440	17.3	80	14.6	67	10.0	94	26.3	19	14.1	180	21.5
General medicine	435	17.1	174	31.7	223	33.3	12	3.4	6	4.4	20	2.4
Radiation oncology	336	13.2	25	4.6	97	14.5	45	12.6	23	17.0	146	17.4
Palliative care	117	4.6	6	1.1	16	2.4	32	8.9	8	5.9	55	6.6
Other*	137	5.4	14	2.6	45	6.7	8	2.2	18	13.3	52	6.2
Missing	205	8.0	43	7.8	31	4.6	22	6.1	21	15.6	88	10.5
**Year of registration**												
2009	177	6.9	3	0.5	15	2.2	40	11.2	11	8.1	108	12.9
2010	875	34.3	110	20.0	179	26.8	131	36.6	53	39.3	402	48.0
2011	712	27.9	196	35.7	209	31.2	88	24.6	30	22.2	189	22.6
2012	785	30.8	240	43.7	266	39.8	99	27.7	41	30.4	139	16.6
**Oncology experience**												
<2 years	1111	43.6	486	88.5	408	61.0	174	48.6	17	12.6	26	3.1
2–5 years	520	20.4	45	8.2	185	27.7	141	39.4	51	37.8	98	11.7
5–10 years	304	11.9	4	0.7	49	7.3	35	9.8	30	22.2	186	22.2
> 10 years	529	20.8	0	0.0	8	1.2	3	0.8	34	25.2	484	57.8
Missing	85	3.3	14	2.6	19	2.8	5	1.4	3	2.2	44	5.3
**Health sector**												
Public	2187	85.8	531	96.7	619	92.5	349	97.5	95	70.4	593	70.8
Private	150	5.9	7	1.3	28	4.2	2	0.6	24	17.8	89	10.6
Both public and private	209	8.2	11	2.0	22	3.3	7	2.0	15	11.1	154	18.4
Missing	3	0.1	0	0.0	0	0.0	0	0.0	1	0.7	2	0.2
**Location**												
NSW	1000	39.2	158	28.8	257	38.4	177	49.4	59	43.7	349	41.6
Other Australian states	1549	60.8	391	71.2	412	61.6	181	50.6	76	56.3	489	58.4
**Recency of use**												
Last 3 months of 2012	1045	41.0	138	25.1	243	36.3	219	61.2	52	38.5	393	46.9
Last 6 months of 2012	1311	51.4	211	38.4	316	47.2	250	69.8	72	53.3	462	55.1
Any time during 2012	1659	65.1	306	55.7	424	63.4	285	79.6	90	66.7	554	66.1
No use in 2012	890	34.9	243	44.3	245	36.6	73	20.4	45	33.3	284	33.9
**Referral source**												
Peer/colleague	1863	73.1	493	89.8	544	81.3	275	76.8	93	68.9	458	54.7
Cancer Institute communication	257	10.1	4	0.7	19	2.8	36	10.1	8	5.9	190	22.7
Conference/booth	49	1.9	3	0.5	12	1.8	6	1.7	5	3.7	23	2.7
Internet	93	3.6	10	1.8	23	3.4	17	4.7	7	5.2	36	4.3
Professional organisation	131	5.1	10	1.8	36	5.4	10	2.8	11	8.1	64	7.6
eviQ education session	102	4.0	23	4.2	25	3.7	9	2.5	6	4.4	39	4.7
Other	54	2.1	6	1.1	10	1.5	5	1.4	5	3.7	28	3.3

*Other: Includes genetics, adolescent, paediatrics.

Doctors were at various stages of oncology training: interns/residents (21.5%), registrars (26.2%), advanced trainees (14.0%), fellows (5.3%), and staff specialists (32.9%) (Table 1). Our cohort worked primarily in medical oncology (34.5%), followed by haematology (17.3%), general medicine (17.1%), and radiation oncology (13.2%); most worked in the public sector (85.8%). Years of oncology experience generally matched level of training; 88.5% of interns had >2 years of oncology experience and 80% of staff specialists had 5–10 or >10 years of oncology experience.

Overall, 65.1% of our cohort had used eviQ at least once during 2012. A greater proportion of advanced trainees (61.2%) and staff specialists (46.9%) used eviQ in the last 3 months of 2012 compared with interns/residents (25.1%), registrars (36.3%), and fellows (38.5%).

The majority of doctors were referred to eviQ by a peer or colleague (73.1% overall); this varied by level of training, ranging from 54.7% (staff specialists) to 89.8% (interns/residents).

#### Patterns of use

##### Webhits by level of training (Figure 2)

**Figure 2:**
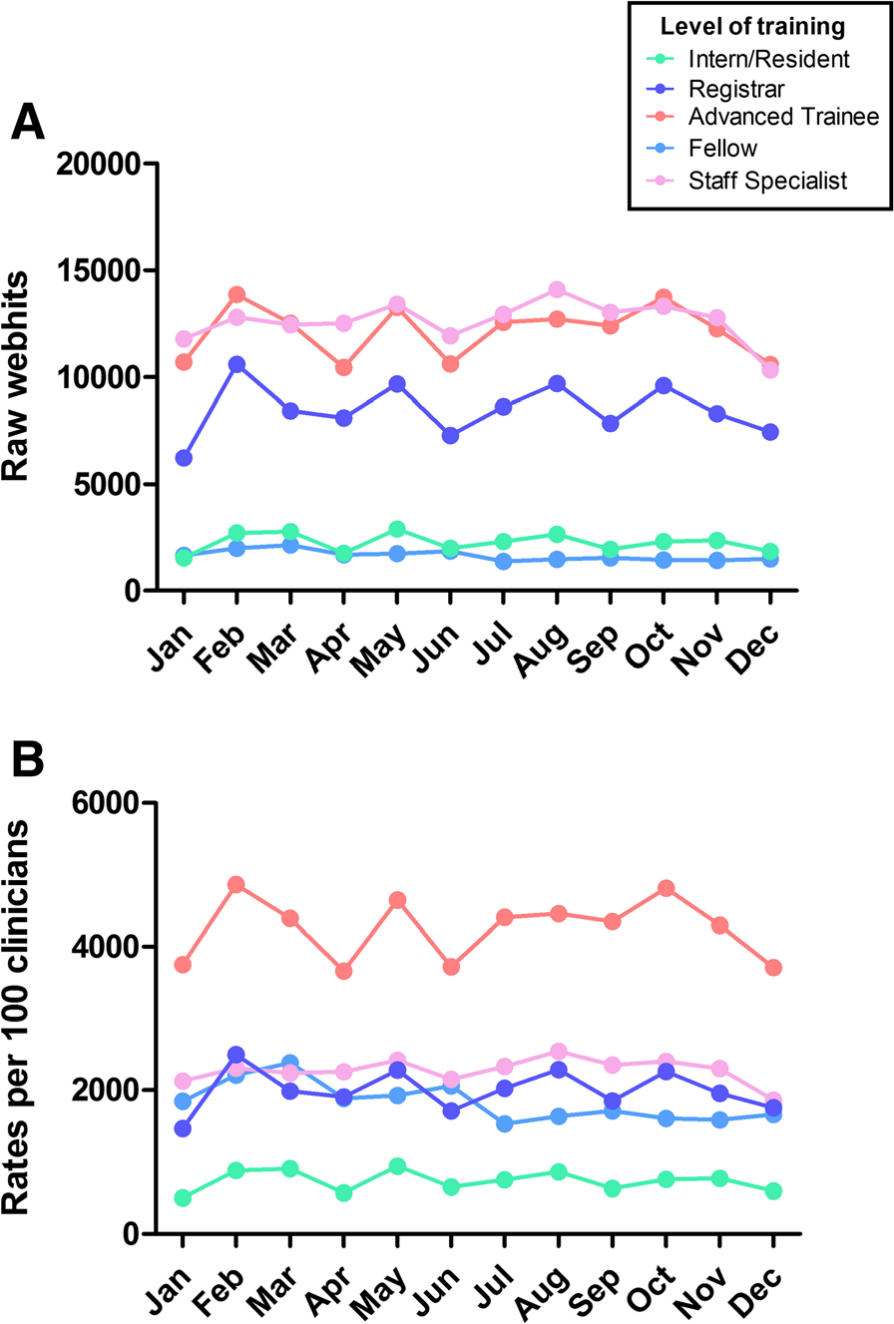
**Webhits by level of training during 2012. A)** Raw webhits; **B)** Rates of use: hits per 100 medical doctors that accessed eviQ during 2012.

Our cohort accounted for 445,492 total webhits in 2012. Staff specialists and advanced trainees had higher numbers of raw webhits compared with doctors at other levels of training; this pattern was consistent across 2012 (Figure 2).

On average, medical doctors had 22.4 hits/month but rates of use varied by level of training, with advanced trainees using eviQ up to five times that of other medical doctors. Specifically, advanced trainees had an average of 42.6 hits/month whereas other groups had fewer hits/month: interns/residents (7.4 hits/month), registrars (20.0 hits/month), fellows (18.4 hits/month) and staff specialists (22.8 hits/month). This pattern was consistent across 2012. Further, based on our previous research demonstrating that 9 pages are viewed during a typical user session [14], we can approximate that on average, advanced trainees use eviQ at least weekly whereas other doctors appear to use the site on a fortnightly or monthly basis.

##### Webhits by years of oncology experience (Figure 3)

**Figure 3:**
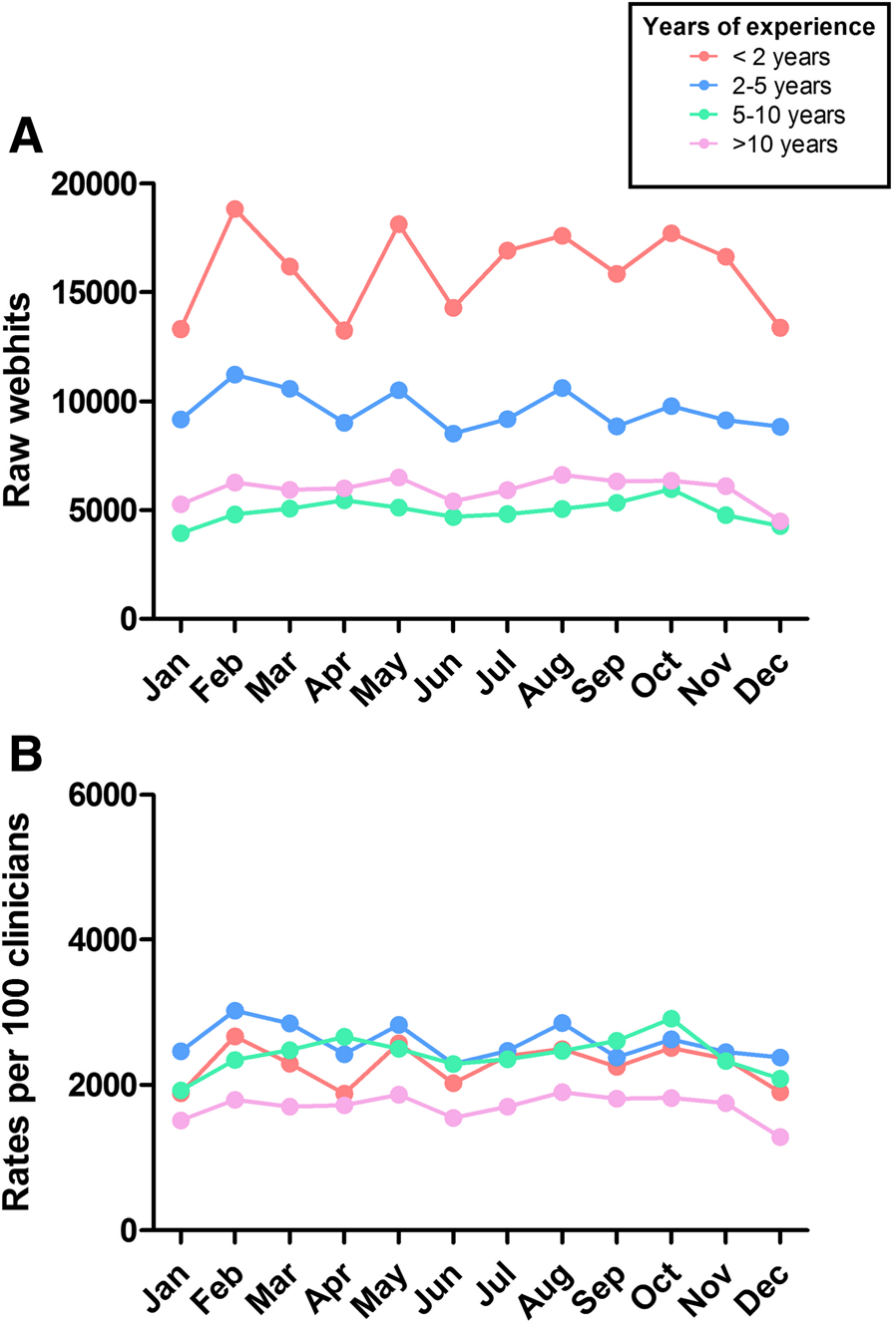
**Webhits by years of oncology experience in 2012. A)** Raw webhits; **B)** Rates of use: hits per 100 medical doctors that accessed eviQ during 2012.

Raw webhits by years of oncology experience were proportional to the size of the clinician groups; clinicians with <2 years of experience accounted for the greatest number of webhits, followed by clinicians with 2–5 years, >10 and 5–10 years of experience; patterns were consistent during the 12 month study period (Figure 3). In contrast to our analysis by level of training, our rate analysis demonstrated less variation in eviQ use by years of oncology experience; mean hits per clinician/month ranged from 17.0 (>10 years’ experience) to 25.9 (2–5 years’ experience) This most likely reflects the fact that professional role (rather than years of oncology experience) determines the nature of doctors’ patient load and thus the utility of eviQ in day-to-day practice. For example, advanced trainees specialising in oncology are likely to see substantially more cancer patients than interns or registrars that rotate across more diverse areas of medicine.

##### Webhits by time of day (Figure 4)

**Figure 4:**
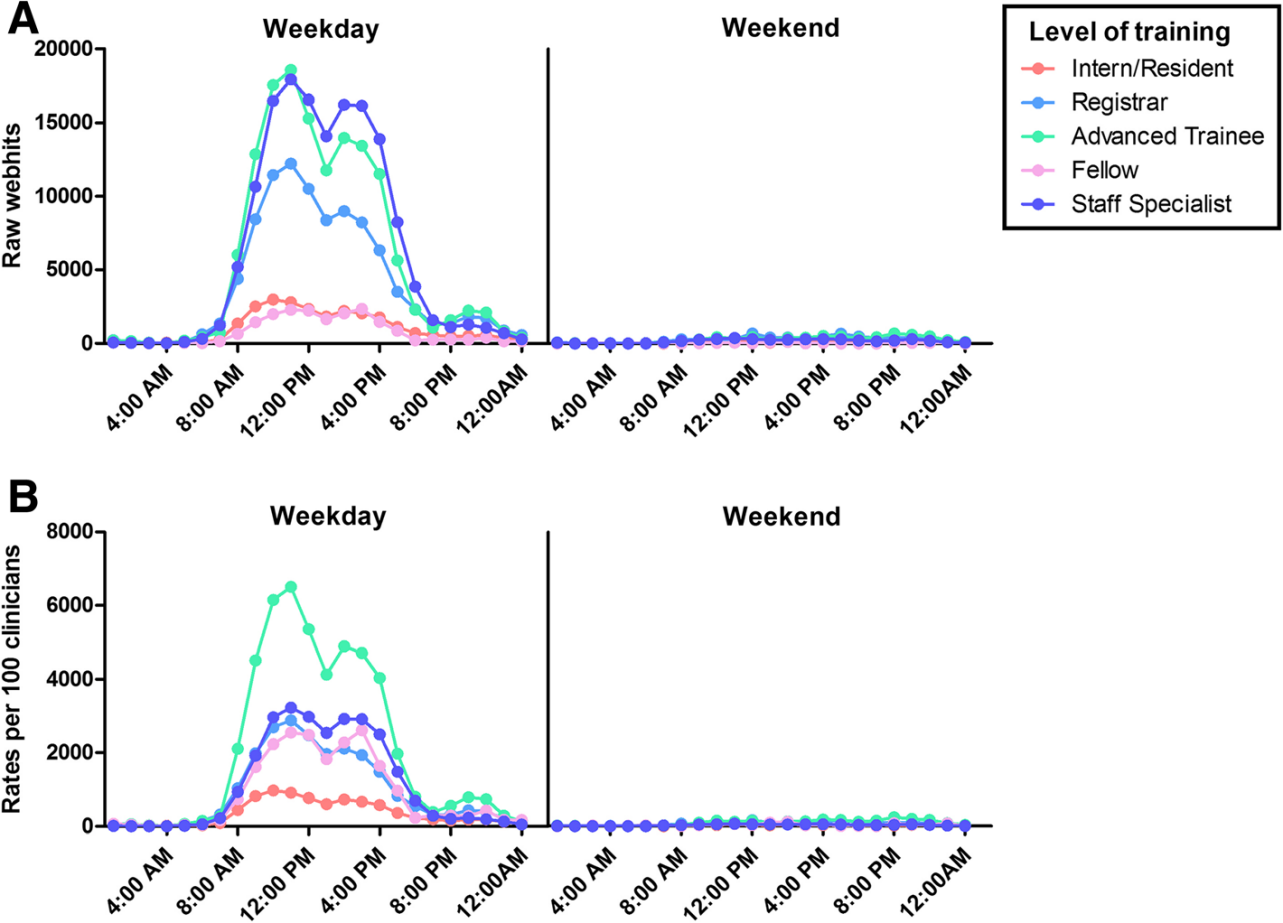
**Webhits by time of day in medical doctors according to level of training in 2012. A)** Raw webhits; **B)** Rates of use: hits per 100 medical doctors that accessed eviQ during 2012.

Our cohort used eviQ primarily during standard clinic hours, with 87.7% of all webhits occurring between 08:00–18:00 Monday to Friday; this pattern of use was similar across all levels of training (Figure 4).

## Phase 2: Online survey of medical doctors

### Methods

#### Study design

Our objective was to compare how eviQ is used by clinicians in training with senior doctors. We developed a 14 item self-report survey to assess doctors’ use of eviQ in their clinical practice. A link to the survey was on the eviQ home page for a 7 week period (September-October 2012); this was accompanied with an invitation to complete the survey. It was estimated that the survey would take no more than 15 minutes to complete and was populated on a server external to eviQ (Google Docs; https://docs.google.com). Upon completion, respondents were invited to send their email address to the researchers to enter a draw to win an Apple iPad; delivered to the winner of the draw in November 2012.

#### Survey respondents

Our target population was Australian medical doctors. As with phase 1, we excluded survey respondents who were general practitioners, students, other health professionals, and those practising outside of Australia.

#### Survey development and content

The survey was developed based on our previous survey work [15,16] and in consultation with the eviQ secretariat and was pilot tested with oncologists and medical students. As such, we established the instrument’s validity but not reliability. The survey consisted of 14 questions; 13 forced choice and one free-text. We collected demographic details, level of training (questions 1–8) and frequency of use of eviQ tools in the past month (e.g., chemotherapy protocols; questions 9–11). We also assessed frequency of eviQ use for clinical tasks (e.g., guiding chemotherapy administration; question 12) and the extent to which respondents agreed with 4 statements about eviQ (e.g., “is integral to my clinical practice”; question 13). Finally, we asked respondents to list any other computer-based systems or websites they use to support their clinical practice. See Additional file 1 for a complete version of the survey.

#### Analysis and reporting

Survey data were analysed using SPSS and Microsoft Excel. We report demographic details and survey responses overall and by level of training.

### Results

#### Characteristics of survey respondents (Table 2)

Of 67 survey respondents, 52 were Australian medical doctors (Table 2). Respondents were at various stages of their training including interns/residents (n = 9, 17.3%), registrars (n = 13, 25.0%), advanced trainees (n = 15, 28.8%), fellows (n=8, 15.4%) and staff specialists (n = 7, 13.5%). Due to small sample sizes, we describe survey results for interns/residents/registrars as one group (n=22) and fellows/staff specialists as one group (n=15). Similar to the characteristics of all medical doctors registered with eviQ (phase 1), most respondents worked in medical oncology (57.7%) and the public sector (94.2%). The majority of respondents used eviQ on a daily basis in the last month (53.8%), the remainder of respondents used eviQ weekly (34.6%) or fortnightly (9.6%).

**Table 2:** Characteristics of survey respondents meeting study eligibility criteria (N = 52)

	**n**	**%**
**Professional role**		
Intern/Resident	9	17.3
Registrar	13	25.0
Advanced trainee	15	28.8
Fellow	8	15.4
Staff specialist	7	13.5
**Oncology experience**		
< 2 years	15	28.8
2–5 years	25	48.1
5–10 years	7	13.5
>10 years	5	9.6
**Location**		
NSW	28	53.8
Other Australian state	24	46.2
**Health sector**		
Public	49	94.2
Private	0	0.0
Both public and private	3	5.8
**Main area of work**		
Medical oncology	30	57.7
Haematology	10	19.2
Radiation oncology	4	7.7
Palliative care	3	5.8
Other*	5	9.6
**eviQ use in the past month**		
Daily	28	53.8
Weekly	5	34.6
Fortnightly	1	9.6
Not at all	18	1.9

*Other: Includes genetics, adolescent, paediatrics.

#### Survey responses (Table 3)

Due to the low survey response rate and small numbers of respondents according to each level of training, we present outcomes for all respondents with some general findings by level of training.

**Table 3:** Survey responses of medical doctors meeting study eligibility criteria (N = 52)

	**Daily**		**Weekly**		**Fortnightly**		**Not at all**	
**In the past month how often did you use the following eviQ tools?**	**n**	**%**	**n**	**%**	**n**	**%**	**n**	**%**
Assessment tools	22	42.3	14	26.9	1	1.9	15	28.8
Cancer genetics	4	7.7	17	32.7	15	28.8	16	30.8
Chemotherapy protocols	26	50.0	15	28.8	9	17.3	2	3.8
Clinical procedures	9	17.3	19	36.5	10	19.2	14	26.9
Patient information	4	7.7	17	32.7	20	38.5	11	21.2
Supportive therapy	4	7.7	13	25.0	20	38.5	15	28.8
Drug calculator	19	36.5	14	26.9	6	11.5	13	25.0
Discussion boards	0	0.0	3	5.8	24	46.2	25	48.1
**In the past month how often have you used eviQ to guide the following?**								
Initiating therapy	16	30.8	25	48.1	7	13.5	4	7.7
Monitoring therapy	2	3.8	13	25.0	23	44.2	14	26.9
Altering therapy	2	3.8	16	30.8	24	46.2	10	19.2
Ceasing therapy	0	0.0	10	19.2	18	34.6	24	46.2
**In the past month how often did you use eviQ to:**								
Compare treatment options	6	11.5	24	46.2	14	26.9	8	15.4
Prescribe medication	8	15.4	18	34.6	14	26.9	12	23.1
Calculate drug dosages	25	48.1	9	17.3	6	11.5	12	23.1
Guide chemotherapy administration	20	38.5	18	34.6	5	9.6	9	17.3
Guide radiotherapy administration	2	3.8	8	15.4	21	40.4	21	40.4
Source treatment cost information	4	7.7	9	17.3	20	38.5	19	36.5
Search for side effects/toxicity	7	13.5	10	19.2	28	53.8	7	13.5
Provide patient information	7	13.5	11	21.2	22	42.3	12	23.1
Teaching resource	5	9.6	11	21.2	15	28.8	21	40.4
Access evidence-based (literature) relevant to practice	11	21.2	25	48.1	9	17.3	7	13.5

##### eviQ tools

The majority of respondents used eviQ tools in the past month with the following tools used on a daily or weekly basis: chemotherapy protocols (88.8%), assessment tools (69.2%), drug calculator (63.4%), and clinical procedures (53.8%) (Table 3). Cancer genetics, patient information, supportive therapy, and discussion boards were the least frequently used tools (6-40% respondents used these tools on a daily/weekly basis).

Advanced trainees reported using eviQ tools more frequently than other medical groups. On average, over 70% of advanced trainees (11/15 respondents) used eviQ tools on a daily/weekly basis compared with less than 30% of fellow/staff specialists (4/15 respondents) and just over half of interns/registrars (13/22 respondents). These results contrast with phase 1, in which interns/residents had the lowest rates of use. However, survey respondents had visited the site in the survey period and were most probably engaged in an oncology rotation at the time. In contrast, the rates of use reported in phase 1 were based on the logfiles of any doctor who had accessed the site in 2012. Interns and residents rotate through different specialties approximately every 10 weeks during their training and it is highly unlikely they would access the site when the rotation is complete.

##### eviQ for clinical tasks

Consistent with its primary purpose, eviQ was most commonly used to initiate therapy (78.9% used eviQ daily or weekly for this task). However, as previously reported [15,16] eviQ was used for the full spectrum of cancer care including monitoring (28.8%), alternating (34.6%), and ceasing therapy (19.2%) on a daily/weekly basis.

eviQ was used frequently (daily/weekly) for a number of specific tasks including: comparing treatment options (57.7%), prescribing medications (50%), calculating drug doses (65.4%), guiding chemotherapy administration (73.1%), and to access evidence-based practice information (69.3%). eviQ was not routinely used to guide radiotherapy administration, access treatment cost information, or as a teaching resource (19.2%, 25.0%, 30.8% reported using eviQ for these tasks on a daily/weekly basis, respectively).

Overall, advanced trainees reported using eviQ more frequently for clinical tasks compared with interns/registrars and staff specialists/fellows; approximately 70% of advanced trainees compared with 50% of interns/registrars and 25% of staff specialists/fellows.

##### Clinicians attitudes towards eviQ

The majority of respondents viewed eviQ favourably. It was endorsed as an up-to-date resource (94.2%), integral to clinical practice (78.9%), and a resource that allows clinicians to function autonomously (80.8%). Further, 67.3% of respondents stated that it was their primary source of oncology information. No respondent 'strongly disagreed' with any statement.

Unlike the frequency of eviQ use for various clinical tasks, clinicians’ attitudes toward eviQ were generally consistent across level of training. Across the four statements, advanced trainees viewed eviQ most favourably (87-100% agreed/strongly agreed), followed by interns/registrars (59-96%), and staff specialists/fellows (53-87%). The greatest variation was in relation to eviQ as a primary information source; 53% of staff specialists/fellows and 59% of interns/registrars compared with 93% of advanced trainees agreed/strongly agreed.

##### Use of other web-based resources

23.1% of respondents listed other online resources they used to support their oncology practice. These included journals/research databases, other oncology websites, and hospital-specific point-of-care programs.

## Discussion

Our study complements a large program evaluation of a web-based oncology protocol system [13-18]. eviQ has been rated among the highest quality online cancer care applications internationally [13,18] and is used at the point-of-care by the key health professionals involved in the delivery of cancer care across Australia [14-16]. In this study, we focus specifically on medical doctors and their utilization of the system across the spectrum of medical training. Our multi-method approach builds upon our previous work and for the first time, allows us to understand the profile of medical doctors using eviQ frequently and intermittently.

Our study suggests that eviQ is an integral part of medical practice in Australia. Importantly, the number of medical oncologists registered on eviQ is similar to that reported in a recent audit of the Australian medical oncology workforce, highlighting the widespread interest in the system in this group of cancer care professionals [20]. Further, given global and local concerns about shortages in the medical oncology workforce [20,21] and clinician burnout [22], systems like eviQ that synthesise the growing evidence-base and streamline clinical practice [15,16] will be increasingly important for the safe and effective delivery of cancer care.

Our results demonstrate important differences in eviQ use according to level of medical training. We found rates of use increase according to level of training and peak with advanced trainees. After doctors are specialty-qualified their rates of use decline to match those of registrars. This is consistent with our previous research that found doctors in training viewed eviQ as important in guiding their clinical practice and for professional autonomy. In contrast, specialty-qualified doctors expressed reservations about routine use of standardised protocols and were less likely to use the system than their more junior colleagues [16].

The different patterns of eviQ use observed in this study directly mirror the roles of clinicians in oncology care at different levels of training. Specifically, interns, residents and registrars rotate across medical specialties and would only generally use eviQ during oncology terms. Advanced trainees plan to specialise in oncology and are likely to have the greatest patient load including direct involvement in initiating and monitoring treatments (under the direct supervision of a specialty-qualified clinician). Finally, staff specialists generally subspecialise in specific cancer treatments (e.g., breast cancer) and become highly familiar with a core group of protocols and treatments. They may only use eviQ when faced with new or unfamiliar clinical situations [15,16]. Nevertheless, the lower rates of use observed in staff specialists may be viewed as less than optimal as speciality-qualified doctors are ultimately responsible for the outcomes of oncology care. Alternatively, our results may indicate that this group of doctors remain up-to-date with latest evidence using other information sources.

Differences in use across levels of training are not unique to online systems in oncology practice. A large-scale evaluation of another Australian evidence-based resource found junior clinicians were more likely to be aware of and use the system at the point-of-care than their senior counterparts [23]. Given that online systems have been found to improve accuracy and confidence of clinicians’ decisions [24,25], reticence on the part of senior doctors may reflect their enduring preference to rely on their own experience or that of their colleagues as their primary information source for point-of-care decisions [26].

Our targeted analysis of doctors’ use of eviQ at various stages in their medical training made use of improvements to the eviQ platform since our previous analyses [14]. Our logfile analysis demonstrated that a significant proportion of medical registrants are not regular visitors to the site. As such, the rate analyses presented in the current study is likely to more accurately reflect current use of the system (22 hits/doctor vs 14 hits/doctor in our previously published logfile analysis). Even with improvements to the capabilities of the eviQ platform, a limitation of our study was the inability of the platform to examine use at an individual doctor level and track this use over time. Clearly, consideration of these issues has important implications for the interpretation of evaluations of this kind.

A further limitation to our current analysis is the lack of specificity of the eviQ platform in identifying years of oncology experience and possible underestimation of use by medical doctors who may have accessed eviQ through oncology unit registrations. We have previously demonstrated that that rates of eviQ use through unit registrations are three times that of individual clinicians [14]. Moreover, we attempted to complement our logfile analyses with an online survey but the low response rates, even when we offered an incentive to respond, highlights the challenge of gaining insights from a group of busy doctors, something that needs to be improved to enhance ongoing monitoring activities. Additionally, our survey only captured responses from doctors that used the website during the 7 week window that the survey was online; as such, it is unlikely that our sample is representative of all medical doctors who use eviQ.

## Conclusion

Electronic decision support systems are undoubtedly the future of medicine [2]. However, the provision of the most state-of-the art system does not guarantee uptake, and this remains an ongoing challenge for contemporary medical practice. An important consideration is that these systems should never replace interactions with colleagues and patients. Rather, system developers, medical educators and health care administrators need to consider how these systems best complement such interactions to enhance medical care. Multi-method evaluation approaches examining end-user attitudes, knowledge and behaviour will add significant value to achieving this goal. Importantly, evaluation should also aim to better understand the way electronic decision support improves real-world processes and outcomes of care to assess the value for money or cost-effectiveness of their deployment.

## Competing interests

The authors declare that they have no competing interests.

## Authors’ contributions

JML contributed to design and conception of the study, data collection and analysis, interpretation and drafting of the manuscript. BB contributed to data collection and analysis, and drafting of the manuscript. NP contributed to data collection and analysis, and drafting of the manuscript. JMP contributed to data analysis and drafting of the manuscript. SP contributed to design and conception of the study, interpretation and drafting of the manuscript. All authors read and approved the final manuscript.

## Pre-publication history

The pre-publication history for this paper can be accessed here:

http://www.biomedcentral.com/1472-6947/13/82/prepub

## Supplementary Material

Additional file 1 Online survey of medical doctors. Click here for file
