# Supplementary material for: How do medical doctors use a web-based oncology protocol system? A comparison of Australian doctors at different levels of medical training using logfile analysis and an online survey
Source: BMC Med Inform Decis Mak. 2013 Aug 4;13:82. doi: 10.1186/1472-6947-13-82 (PMC3750334; doi:10.1186/1472-6947-13-82)
Supplement: Additional file 1 — Online survey of medical doctors. [file 1472-6947-13-82-S1.pdf]

Additional File 1-survey

Survey: How do you use eviQ?
If you are currently working in oncology as a medical doctor, we invite you to complete a short survey (5-10 minutes) that aims to better understand the way in which doctors use eviQ™® in their daily practice.
THIS SURVEY IS INDEPENDENT FROM THE CANCER INSTITUTE OF NEW SOUTH WALES.
Upon completion, you will have the option to enter a draw to win an iPad by sending an email to the research team; however this personal information will NOT be linked to your survey responses.
Your completion of this survey will indicate your consent to participate in the research.
This research has been approved by the Population and Health Services Research Ethics Committee.
Please contact Dr Julia Langton, Faculty of Pharmacy, The University of Sydney, by phone (02) 9036 9369 or email julia.langton@sydney.edu.au if you would like any further information about this research.

1) What is your primary role:
a) Medical
b) Nursing
c) Radiation Therapy
d) Pharmacy
e) Other: __________

2) What is your area of work:
a) Clinical
b) Industry
c) Management
d) Research
e) Other: __________

3) What is your professional level:
a) Advanced Trainee
b) Fellow
c) General Practitioner
d) Intern/Resident
e) Registrar
f) Staff Specialist
g) Other

4) What is your main area of work:
a) Adolescent/Paediatrics
b) General Medicine
c) General Practice
d) Genetics
e) Haematology
f) Medical Oncology
g) Palliative Care
h) Radiation Oncology
i) Other

5) How many years of professional experience have you had in oncology:
a) Less than 2 years

b) Between 2 to 5 years
c) Between 5 to 10 years
d) More than 10 years
e) Other: __________

6) Which Australian state are you working in: (Please select other if you are working outside of Australia)
a) Australian Capital Territory
b) New South Wales
c) Northern Territory
d) Queensland
e) South Australia
f) Tasmania
g) Victoria
h) Western Australia
i) Other: __________

7) Are you working in primary health care: (Please select "no" if you are working in a hospital setting)
a) Yes
b) No

8) Do you work in the public or private sector:
a) Public
b) Private
c) Both
d) Other: __________

9) In the past month, how often did you use eviQ:
a) Daily
b) Weekly
c) Fortnightly
d) Never

10) In the past month, how often did you use the following eviQ tools:

	Daily	Weekly	Fortnightly	Not at all
Assessment tools				
Cancer genetics				
Chemotherapy protocols				
Clinical procedures				
Patient information				

Supportive therapy				
Drug calculator				
Discussion Boards				

11) In the past month, how often did you use eviQ to guide the following:

	Daily	Weekly	Fortnightly	Not at all
Initiating therapy				
Monitoring therapy				
Altering therapy				
Ceasing therapy				

12) In the past month, how often did you use eviQ to:

	Daily	Weekly	Fortnightly	Not at all
Compare treatment options				
Prescribe medication				
Calculate drug doses				
Guide chemotherapy administration				
Guide radiotherapy administration				
Source treatment cost information				
Search for side effects/toxicity				
Provide patient				

information				
Teaching Resource				
Access evidence-base (e.g., journal articles) relevant to practice				

13) To what extent do you agree with the following statements:

	Strongly Disagree	Disagree	Neither agree nor disagree	Agree	Strongly Agree
eviQ……..	**1**	**2**	**3**	**4**	**5**
is integral to my clinical practice					
allows me to function independently as a clinician					
is an up-to-date source of cancer treatment information					
is my primary source of oncology treatment information					

14) Please list any other computer-based systems or websites you use to support your clinical practice:
